# Phenotypic Variation in Mangrove Cuckoo (*Coccyzus minor*) across Its Geographic Range

**DOI:** 10.1371/journal.pone.0152141

**Published:** 2016-03-23

**Authors:** John D. Lloyd

**Affiliations:** 1 Vermont Center for Ecostudies, Norwich, Vermont, United States of America; 2 Ecostudies Institute, Mount Vernon, Washington, United States of America; Università degli Studi di Milano-Bicocca, ITALY

## Abstract

Mangrove Cuckoo (*Coccyzus minor*) exhibits substantial phenotypic variation across its geographic range, but the significance of this variation for taxonomy remains unresolved. Using measurements of bill size and ventral color recorded from 274 museum specimens, I found that variation in these traits was clinal. No named subspecies was reciprocally diagnosable from all others, and none was distinguishable from the nominate form, such that previously recognized subspecific distinctions are invalid. Greatest differences in phenotype occurred between populations in Florida, the Bahamas, and the Greater Antilles–characteristically small-billed–and those in the Lesser Antilles, which had larger bills. Phenotypically intermediate individuals on the geographically intermediate islands of Barbuda and Antigua linked these two extremes. Individuals intermediate in bill size and color also characterized populations from throughout the remainder of the range in northern South America and Middle America. Mechanisms maintaining the fairly pronounced phenotypic differences between nearby populations of Greater and Lesser Antillean birds are unknown, yet the geographic proximity of these populations suggests that they probably persist despite occasional gene flow, and may be adaptive.

## Introduction

Mangrove Cuckoo (*Coccyzus minor*) exhibits extensive variation in phenotype, especially in bill size, bill shape, and color of the ventral plumage [1]. Making sense of this variation has proven difficult. One approach has been to classify populations into recognizable subspecies, an effort that, at its zenith, yielded 14 named forms [2]. However, this approach was criticized in a subsequent review of the taxonomy of Mangrove Cuckoo, which, based on visual inspection of a series of specimens, argued that all phenotypic variation in the species was random with respect to geography [3]. Until the question could be answered definitively by a quantitative analysis of a larger sample of individuals, Banks & Hole [3] recommended recognizing no subspecies. Accordingly, most avian taxonomies (e.g., [4]) now treat the species as monotypic, although questions persist about whether some populations may indeed be distinctive enough to warrant recognition as subspecies [5].

Here, in an effort to to resolve lingering questions about phenotypic variation in Mangrove Cuckoo and its taxonomic implications, I describe geographic patterns in phenotype and then provide the first formal analysis of diagnosability of putative subspecies. Despite the long history of debate concerning the validity of different subspecies of Mangrove Cuckoo, geographic patterns of variation in appearance remain largely unexplored. Generating a better understanding of the nature of phenotypic variation in this species is important not only because it underpins our understanding of infraspecific taxonomy, but because it has been used to draw inference about the natural history of Mangrove Cuckoo. For example, the lack of any consistent pattern in phenotype was used to infer that individuals were highly vagile; that long-distance dispersal events, perhaps driven by tropical cyclones, were relatively common and played a significant role in determining the geographic range of the species; and that local adaptation played no significant role in shaping the evolution of traits such as bill size and plumage color [3]. Mangrove Cuckoos are poorly studied and their ecology largely unknown [5,6], and these inferences still represent a large part of our current understanding of this species.

## Materials and Methods

I collected data on morphology and plumage color for 12 of the 14 subspecies listed by Peters [2], which was the most recent major treatment of subspecific variation in Mangrove Cuckoo. The two subspecies that I did not include in this analysis consisted of one (*C*. *m*. *ferrugineus*) that is now recognized as a distinct species (*C*. *ferrugineus*) [7] and one (*C*. *m*. *cozumelae*) for which I could not acquire specimens. *Coccyzus minor cozumelae*, known from only 3 specimens, was the last subspecies described [8] and was quickly synonymized with *C*. *m*. *continentalis* by Paynter [9], who called it “untenable” as a distinct subspecies. For the 12 other subspecies, I measured specimens held at four museums with major collections of Mangrove Cuckoo: American Museum of Natural History, National Museum of Natural History at the Smithsonian Institution, Field Museum of Natural History, and the Museum of Comparative Zoology. I recorded information on sex, date of collection, and collection location from the specimen tag. I assigned each specimen to a subspecies by comparing the collection location to the geographic distribution for each subspecies as reported by Peters [2]. In cases where two putative subspecies were reported to have sympatric distributions (e.g., *C*. *m*. *nesiotes* and *C*. *m*. *teres*), I used the identification provided by the collector. Mangrove Cuckoo appears to be a year-round resident throughout its range [6], so I assumed that specimens belonged to the breeding population at the collection locale. All data used in this analysis, including all specimen numbers and the name of the permanent repository for each specimen, are available at http://dx.doi.org/10.6084/m9.figshare.1328170. No permits were required for the described study, which complied with all relevant regulations.

Bill size and color of the ventral plumage were the diagnostic features most commonly referred to in the original descriptions of each subspecies (e.g., see [1] for a description of variation in these two traits across subspecies), so I focused on these traits. Earlier authors were vague on exactly which elements of bill size they considered, using adjectives such as “heavy”, “weaker”, “stouter”, “larger”, and “smaller” (e.g., [10,11]). As such, I measured bill length (anterior edge of the nares to tip of the bill), bill width (width of the bill at the anterior edge of the nares), and bill depth (depth of the bill at the anterior edge of the nares). I used dial calipers and recorded measurements to the nearest 0.1 mm.

Plumage color on the ventral surface of Mangrove Cuckoo ranges from a creamy white to a tawny buff, with the richest colors generally found posteriorly and the lightest colors from the breast to the chin [6]. I recorded color in four regions of the ventral surface: vent, belly, breast, and chin. In order to visually isolate the color in each region, I placed a white piece of paper over the region and recorded color in a 1-cm^2^ square opening cut in the paper. On each specimen, I scored color in each region using a 5-point categorical scale based on the range of variation that I observed during an initial assessment of 138 specimens, which included representatives of each subspecies. The lightest color (scored as 0) was equivalent to the 4A2 Methuen standard [12], and the darkest color that I observed (scored as 5) was equivalent to the 5B5 Methuen standard.

I estimated the repeatability of bill measurements and color scoring by decomposing the sources of variation in a one-way analysis of variance [13]. I applied this method to a random sample of 17 individuals that I measured and scored twice. Individuals included in the sample represented seven subspecies: *C*. *m*. *continentalis* [n = 5], *C*. *m*. *vincentis* [n = 3], *C*. *m*. *maynardi* [n = 3], *C*. *m*. *teres* [n = 2], *C*. *m*. *grenadensis* [n = 3], *C*. *m*. *dominicae* [n = 1], and *C*. *m*. *abbotti* [n = 1]. To avoid biasing my estimates of repeatability, I never took a second series of measurements on an individual immediately after recording its initial measurements; I measured all other individuals in the collection before re-measuring any individual.

Although Mangrove Cuckoo is considered sexually monomorphic, I used multivariate analysis of variance to test for differences between males and females in bill size and ventral color while controlling for differences among putative subspecies. I screened for outliers and addressed the assumption of multivariate normality by plotting Mahalanobis distances against estimated quantiles from a chi-square distribution.

I examined geographic patterns in the three measures of bill size with cluster analysis, using the R package NbClust [14] to identify the optimal number and partitioning of clusters. Clusters were generated using the complete linkage method applied to Euclidean distances. I summarized univariate patterns in bill depth, bill width, and bill length using both discriminant function analysis (DFA) and principal components analysis (PCA). I used the R package vegan [15] to execute the PCA and the R package MASS [16] for DFA.

I calculated pairwise diagnosability indices [17] for the composite measure of bill size generated by the PCA. By applying the diagnosability test to the composite measure of bill size, rather than to any single measure of bill size, I sought to closely align my test of the validity of the named subspecies with the original descriptions, which often relied on qualitative assessments of overall bill size. My application of Patten and Unitt’s [17] diagnosability index implements the “75% rule” [18], which defines a valid subspecies as one in which 75% of the distribution of values for some character in one population falls outside the range of values for that same character in another population. Although other criteria exist, most of which set a higher threshold (e.g., [19]), the 75% rule is a widely adopted basis for defining valid subspecies [17]. A pairwise diagnosability value ≥ 0 indicates that two putative subspecies can be distinguished under this rule, and the test must be positive in both directions (i.e., from taxa A to taxa B, and from taxa B to taxa A) because diagnosability is not necessarily symmetric. I also applied the 75% rule directly to the observed distributions of ventral color scores in order to determine if subspecies were diagnosable based on this trait.

## Results

**Fig 1:**
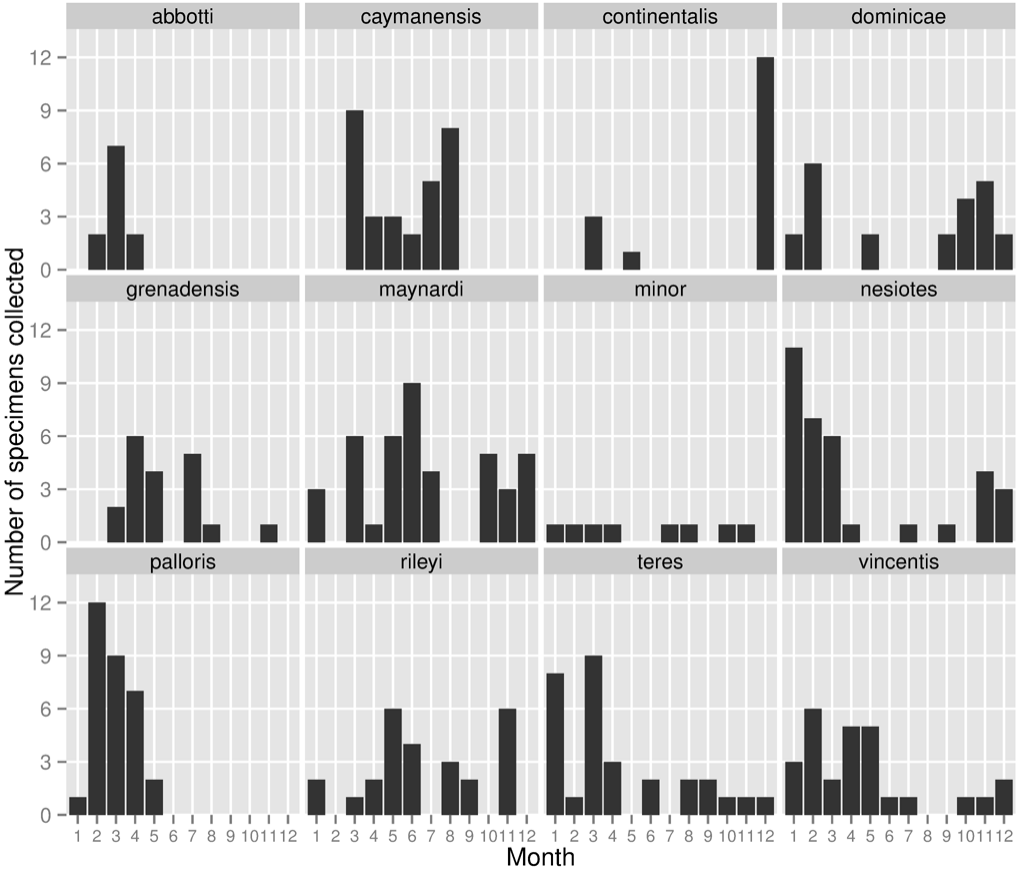
Date of collection for specimens of Mangrove Cuckoo (*Coccyzus minor*). The time of year in which specimens were collected varied among the different subspecies of Mangrove Cuckoo.

I measured a total of 274 individuals from the 12 subspecies for which specimens were available (Table 1). Collection dates varied widely across subspecies and for most subspecies were not distributed evenly across the year (Fig 1). Several of the subspecies examined, such as *C*. *m*. *abotti* or *C*. *m*. *continentalis*, were represented by specimens collected during a small number of expeditions and as such had a very narrow range of collection dates.

**Table 1:** Number of specimens measured and geographic range of each named subspecies of Mangrove Cuckoo (*Coccyzus minor*) included in this analysis.

Named subspecies	Number of specimens	Geographic range
*C*. *m*. *abbotti*	10	Providencia, San Andrés
*C*. *m*. *caymanensis*	25	Cayman Islands
*C*. *m*. *continentalis*	18	Caribbean slope of Middle America
*C*. *m*. *dominicae*	27	Montserrat, Guadeloupe, Dominica
*C*. *m*. *grenadensis*	21	Grenada, Grenadines
*C*. *m*. *maynardi*	32	Florida (USA), Bahamas
*C*. *m*. *minor*	8	Northern South America
*C*. *m*. *nesiotes*	30	Greater Antilles
*C*. *m*. *palloris*	30	Pacific slope of Middle America
*C*. *m*. *rileyi*	23	Barbuda, Antigua
*C*. *m*. *teres*	27	Greater Antilles (except Jamaica and Cuba)
*C*. *m*. *vincentis*	23	Martinique, St. Lucia, St. Vincent

Neither bill size nor ventral color differed significantly between male and female specimens (bill size: Pillai’s trace = 0.01, approximate F = 0.49, P = 0.74; ventral color: Pillai’s trace = 0.02, approximate F = 1.37, P = 0.24), so I pooled sexes for all subsequent analyses. Repeatability of measurements of bill depth (0.64), bill width (0.67), and bill length (0.81) was moderate to high [20]. The maximum difference in repeated measures was 0.2 mm (mean = 0.09) for bill depth, 0.7 mm (mean = 0.31) for bill width, and 0.5 mm (mean = 0.13) for bill length. Repeatability of the four color measurements was consistently high (all > 0.9).

**Fig 2:**
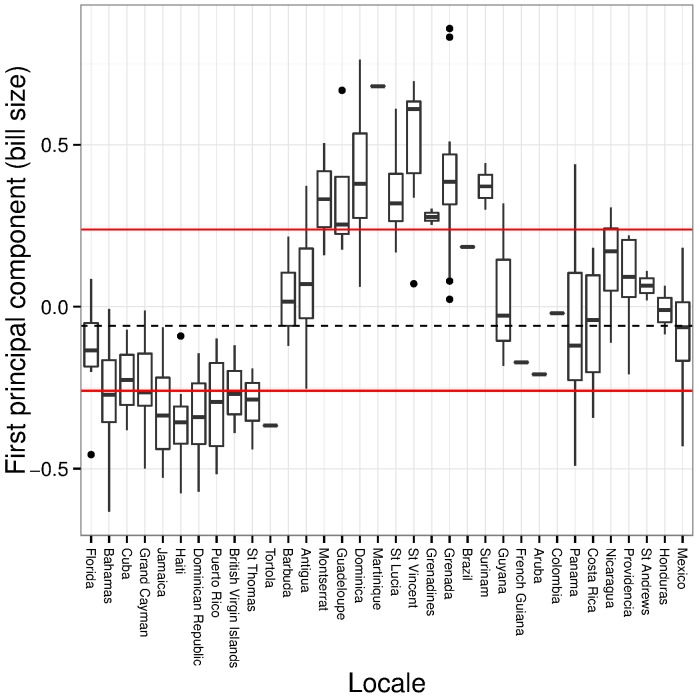
Bill size of Mangrove Cuckoo (*Coccyzus minor*). A composite measure of bill size (PC1; the first principal component from an analysis of bill length, bill depth, and bill width) of Mangrove Cuckoo subspecies varied clinally. Boxplots for each collection locale are arranged in geographic order beginning with the northernmost populations (Florida) and proceeding clockwise around the Caribbean Basin.

The three univariate measures of bill size varied concordantly: the first principal component in the PCA accounted for 71.4% of the explained variation, and was positively associated with bill depth (*r* = 0.62), bill width (*r* = 0.55), and bill length (*r* = 0.56). The first principal component thus described a general measure of bill size. Bill size was similar among geographically proximate populations, but varied substantially among broader geographic regions (Fig 2). Relatively small-billed birds in Florida, the Bahamas, and Greater Antilles were separated from the relatively large-billed populations in the Lesser Antilles by the phenotypically intermediate populations on Antigua and Barbuda, which in turn were similar in bill size to populations throughout South and Middle America (Fig 2). Large-billed individuals, like those characteristic of birds from Montserrat to Grenada, were notably absent from collections made in Florida, the Bahamas, or the Greater Antilles.

A similar pattern was recovered by cluster analysis of the three univariate measures of bill size. The optimal number of clusters was two and reflected the division between large-billed birds of the Lesser Antilles and small-billed birds from the Greater Antilles and Florida and the Bahamas. The first cluster included all specimens from the Greater Antilles and all but one of the specimens from Florida and the Bahamas, whereas the second cluster contained nearly all of the specimens from the Lesser Antilles (92%). Specimens from the remaining regions were split evenly between the two clusters (Antigua and Barbuda: first cluster, n = 15; second cluster, n = 8; Middle America: first cluster = 32; second cluster = 23; South America: first cluster, n = 5; second cluster, n = 4).

**Fig 3:**
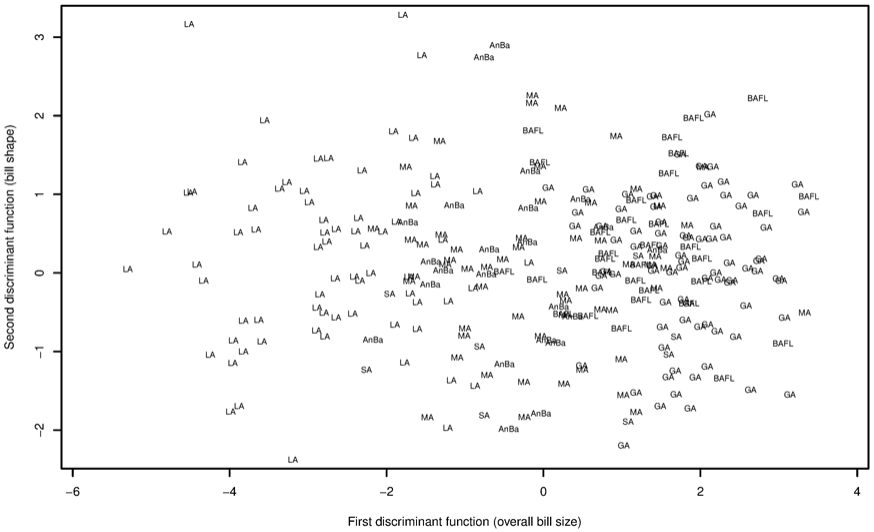
Visualization of discriminant function analysis of bill size of Mangrove Cuckoo (*Coccyzus minor*) specimens. The first function in a discriminant function analysis of three components of bill size (bill depth, bill width, and bill length) clearly separated large-billed specimens of Mangrove Cuckoo of the Lesser Antilles (LA) from small-billed specimens collected in the Bahamas and Florida (BAFL) and the Greater Antilles (GA). Specimens from Antigua and Barbuda (AnBa), South America (SA), and Middle America (MA) were intermediate in bill size.

A discriminant function analysis of all three components of bill size clearly separated large-billed birds of the Lesser Antilles from the small-billed birds of Florida, the Bahamas, and the Greater Antilles (Fig 3). The first discriminant axis, which accounted for nearly all of the explained between-group variance (91%), was essentially a measure of overall bill size, but was most strongly associated with bill depth (first discriminant function coefficients: bill depth = -1.9, bill width = -0.35, and bill length = -0.31). The second discriminant axis, which accounted for relatively little between-group variance (7%), provided a measure of bill shape, distinguishing specimens with relatively wide but shallow bills from those with narrower and deeper bills. Correct classification rates based on the discriminant functions reinforced patterns evident from the cluster analysis: specimens from the Bahamas, Florida, and the Greater Antilles were indistinguishable from one another, but were readily separable from specimens collected in the Lesser Antilles (Table 2). Specimens from other regions were frequently misclassified, but largely without any evident geographic patterns; one notable exception were specimens from Antigua and Barbuda, which were frequently misclassified as South or Middle American in origin but rarely misclassified as deriving from the Greater or Lesser Antilles.

**Table 2:** Proportion of specimens of Mangrove Cuckoo (*Coccyzus minor*) assigned by a discriminant function analysis to each geographic region from which specimens were collected. Proportion of correct classifications are shown in bold along the diagonal.

	Predicted region of collection					
Actual region of collection	Florida and the Bahamas	Greater Antilles	Antigua and Barbuda	Lesser Antilles	South America	Middle America
Florida and the Bahamas	**0.41**	0.34	0.09	0.00	0.06	0.09
Greater Antilles	0.24	**0.63**	0.01	0.00	0.05	0.06
Antigua and Barbuda	0.04	0.00	**0.35**	0.09	0.30	0.22
Lesser Antilles	0.00	0.00	0.07	**0.83**	0.04	0.06
South America	0.11	0.22	0.00	0.22	**0.33**	0.11
Middle America	0.14	0.05	0.14	0.11	0.23	**0.33**

**Fig 4:**
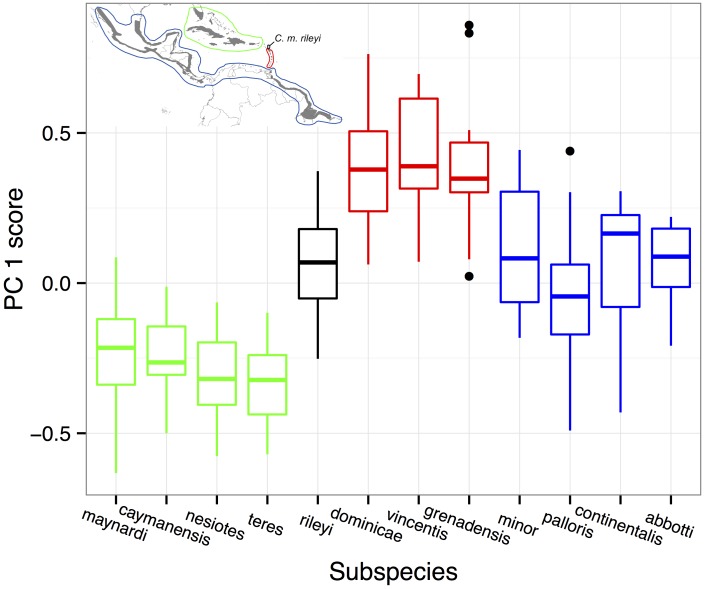
Bill size of Mangrove Cuckoo (*Coccyzus minor*). A composite measure of bill size (PC1; the first principal component from an analysis of bill length, bill depth, and bill width) of Mangrove Cuckoo subspecies varied along a geographic cline. Subspecies are arranged in geographic order, and colored by region (see inset map).

Similar patterns emerged when comparing bill size among historically recognized subspecies. Bill size was smallest among birds from subspecies in Florida and the Bahamas and the Greater Antilles, largest among the Lesser Antillean subspecies, and intermediate in the South and Middle American subspecies (Fig 4). Subspecies from Florida, the Bahamas, and the Greater Antilles had almost no overlap in bill size with the Lesser Antillean subspecies, but were linked by the geographically and morphologically intermediate *C*. *m*. *rileyi* from the islands of Antigua and Barbuda

The DFA treating subspecies as the grouping factor produced nearly identical results as the DFA treating different regions as groups. Almost all the between-class variance was described by the first discriminant function (90.5%), which was a measure of bill size driven primarily by differences in bill depth (first discriminant function coefficients: bill depth = 2.03, bill width = 0.39, and bill length = 0.24). Most specimens were misclassified to subspecies by this function (Table 3). Misclassifications were most common among the subspecies from Florida and the Bahamas and the Greater Antilles and among the subspecies of the Lesser Antilles. In contrast, predictions from the first discriminant function rarely confused the large-billed *C*. *m*. *dominicae* and *C*. *m vincentis* with any of the smaller-billed forms of the Greater Antilles, or vice versa. The named subspecies from South America and Middle America were frequently misclassified, but without any clear geographic pattern.

**Table 3:** Proportion of specimens of Mangrove Cuckoo (*Coccyzus minor*) assigned by a discriminant function analysis to each of 12 named subspecies. Proportion of correct classifications are shown in bold along the diagonal.

	Predicted identity											
Actual identity	*maynardi*	*caymanensis*	*nesiotes*	*teres*	*rileyi*	*dominicae*	*vincentis*	*grenadensis*	*minor*	*continentalis*	*palloris*	*abbotti*
*maynardi*	**0.31**	0.09	0.09	0.19	0.06	0.00	0.00	0.00	0.00	0.06	0.13	0.06
*caymanensis*	0.16	**0.08**	0.20	0.28	0.00	0.00	0.00	0.00	0.08	0.00	0.20	0.00
*nesiotes*	0.03	0.03	**0.60**	0.23	0.00	0.00	0.00	0.00	0.03	0.00	0.07	0.00
*teres*	0.15	0.00	0.37	**0.41**	0.04	0.00	0.00	0.00	0.00	0.00	0.04	0.00
*rileyi*	0.00	0.04	0.00	0.00	**0.30**	0.09	0.04	0.00	0.22	0.00	0.22	0.09
*dominicae*	0.00	0.00	0.00	0.00	0.07	**0.48**	0.30	0.07	0.07	0.00	0.00	0.00
*vincentis*	0.00	0.00	0.00	0.00	0.04	0.52	**0.26**	0.04	0.09	0.00	0.04	0.00
*grenadensis*	0.00	0.00	0.00	0.00	0.05	0.14	0.10	**0.67**	0.05	0.00	0.00	0.00
*minor*	0.13	0.00	0.13	0.00	0.00	0.00	0.25	0.00	**0.38**	0.00	0.13	0.00
*continentalis*	0.00	0.06	0.00	0.11	0.11	0.11	0.00	0.22	0.28	**0.06**	0.06	0.00
*palloris*	0.13	0.00	0.03	0.03	0.10	0.03	0.00	0.03	0.10	0.03	**0.47**	0.03
*abbotti*	0.00	0.00	0.10	0.00	0.30	0.00	0.00	0.00	0.30	0.10	0.10	**0.10**

**Fig 5:**
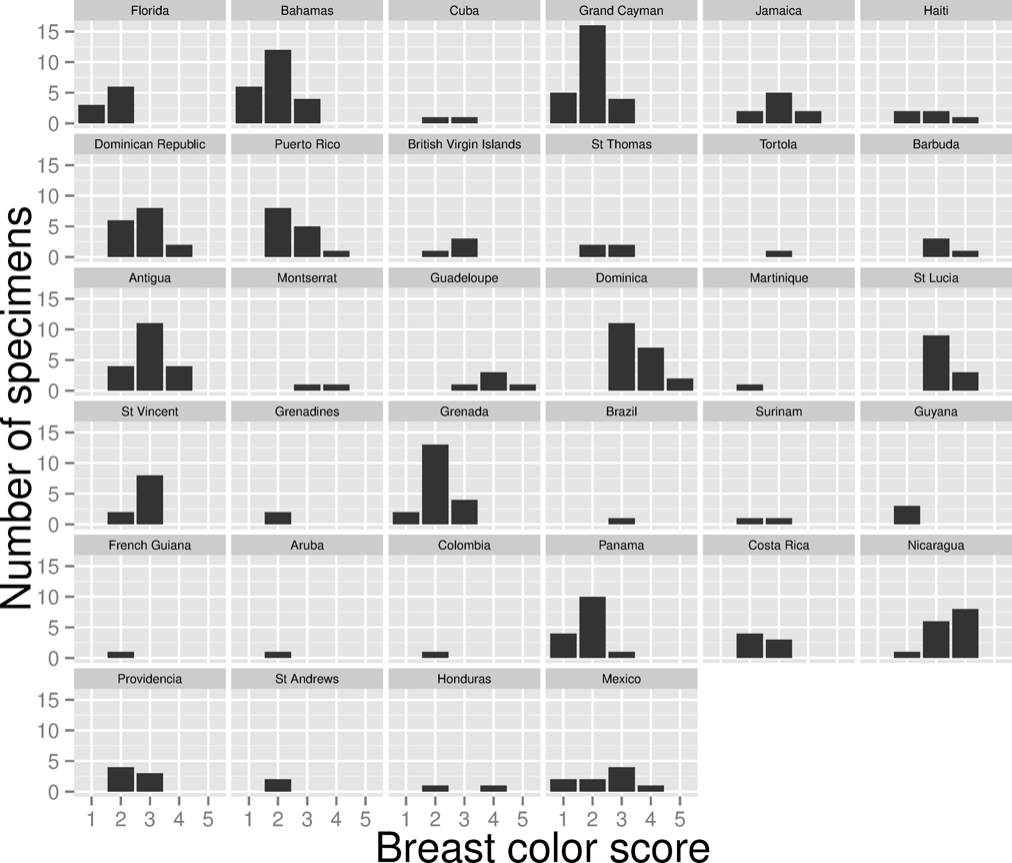
Breast plumage color of of Mangrove Cuckoo (*Coccyzus minor*). Color of the breast plumage (scored from 1–5, with 1 being lightest and 5 darkest) of Mangrove Cuckoos collected in different locations. Relatively pale specimens characterized populations from Florida, the Bahamas, the Cayman Islands, Grenada, South America, and some regions of Middle America (especially the Pacific slope of Panama). Relatively dark individuals were most common in the Lesser Antilles, especially Dominica, and the Greater Antilles.

**Fig 6:**
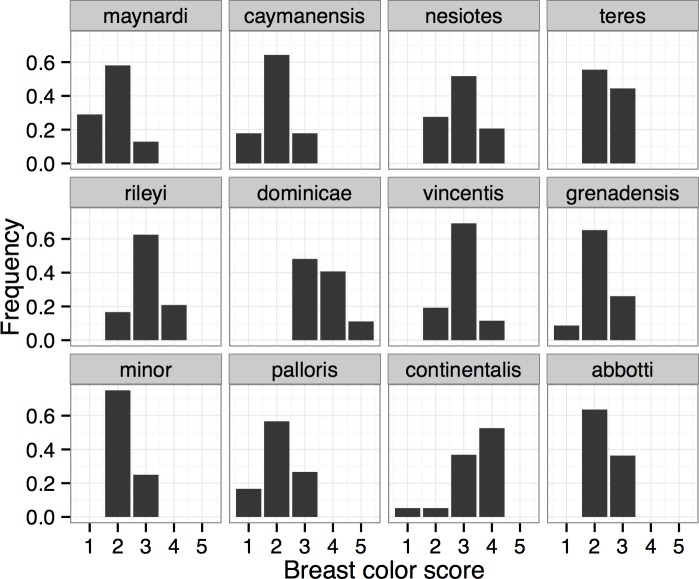
Breast plumage color of named subspecies of Mangrove Cuckoo (*Coccyzus minor*). Color of the breast plumage (scored from 1–5, with 1 being lightest and 5 darkest) of Mangrove Cuckoo subspecies. Subspecies are arranged in geographic order from the upper left panel. Relatively pale specimens characterized populations from Florida and the Bahamas (*C*. *m*. *maynardi*), the Cayman Islands (*C*. *m*. *caymanensis*), Grenada (*C*. *m*. *grenadensis*), South America (*C*. *m*. *minor*), and the Pacific Slope of Middle America (*C*. *m*. *palloris*). Relatively dark individuals characterized other populations in the Greater (*C*. *m*. *nesiotes*, *C*. *m*. *teres*, and *C*. *m*. *rileyi*) and Lesser Antilles (*C*. *m*. *dominicae* and *C*. *m*. *vincentis*), as well as individuals from the Caribbean slope of Middle America and its adjacent islands (*C*. *m*. *continentalis* and *C*. *m*. *abbotti*).

Ventral color varied concordantly among the four body regions (Spearman rank-correlation coefficients of scores at different regions ranged from 0.58 to 0.82). For brevity I present only information on breast color as this character was more variable than chin, vent, or belly color. The geographic pattern of variation in breast color was not as consistent as that observed for bill size, although a cline from light-colored birds in Florida, the Bahamas, and Cayman Islands to very dark birds in the central Lesser Antilles was evident (Fig 5). Pale specimens characterized subspecies at both ends of the geographic range: birds from Florida, the Bahamas, and Cayman Islands were relatively pale, as were birds from Grenada, northern South America, and the Pacific slope of Middle America (Fig 6; S1 Photograph). Other populations in the Greater and Lesser Antilles were relatively dark, as were birds from the Caribbean slope of Middle America (including *C*. *m*. *abbotti*). The richest ventral color was expressed among specimens of *C*. *m*. *dominicae* (Fig 6; S1 Photograph). Despite these general patterns of variation in color, exceptions were common (e.g., some individuals from the typically pale *C*. *m*. *maynardi* were as dark as individuals from the darkest named subspecies, *C*. *m*. *dominicae*; S2 Photograph).

Each of the subspecies from Florida and the Bahamas and the Greater Antilles was reciprocally diagnosable from each of the three Lesser Antillean subspecies based on bill size (Table 4), with the exception of *C*. *m*. *grenadensis*, which could not be diagnosed from *C*. *m*. *maynardi*. None of the other named subspecies–including the nominate *C*. *m*. *minor*–were diagnosable from one another under the 75% rule applied in this analysis.

**Table 4:** Pairwise diagnosability indices of bill size for 12 named subspecies of Mangrove Cuckoo (*Coccyzus minor*).

	Named subspecies											
	*maynardi*	*caymanensis*	*nesiotes*	*teres*	*rileyi*	*dominicae*	*vincentis*	*grenadensis*	*minor*	*continentalis*	*palloris*	*abbotti*
*maynardi*	-	-0.41	-0.38	-0.34	-0.22	**0.00**	**0.10**	-0.01	-0.46	-0.37	-0.40	-0.27
*caymanensis*	-0.50	-	-0.35	-0.30	-0.18	**0.04**	**0.13**	**0.02**	-0.43	-0.34	-0.36	-0.23
*nesiotes*	-0.44	-0.32	-	-0.38	-0.37	**0.09**	**0.19**	**0.08**	-0.37	-0.28	-0.31	-0.18
*teres*	-0.41	-0.29	-0.39	-	-0.09	**0.13**	**0.22**	**0.11**	-0.34	-0.25	-0.27	-0.14
*rileyi*	-0.23	-0.11	-0.08	-0.03	-	-0.28	-0.19	-0.30	-0.75	-0.65	-0.50	-0.54
*dominicae*	**0.07**	**0.19**	**0.22**	**0.27**	-0.20	-	-0.55	-0.65	-0.55	-0.39	-0.20	-0.24
*vincentis*	**0.13**	**0.25**	**0.28**	**0.33**	-0.14	-0.59	-	-0.59	-0.49	-0.33	-0.14	-0.18
*grenadensis*	**0.06**	**0.18**	**0.21**	**0.26**	-0.21	-0.64	-0.55	-	-0.55	-0.40	-0.20	-0.25
*minor*	-0.24	-0.12	-0.09	-0.05	-0.51	-0.39	-0.30	-0.55	-	-0.70	-0.51	-0.55
*continentalis*	-0.26	-0.14	-0.28	-0.06	-0.53	-0.34	-0.25	-0.36	-0.81	-	-0.52	-0.56
*palloris*	-0.35	-0.23	-0.20	-0.15	-0.43	-0.22	-0.12	-0.23	-0.68	-0.59	-	-0.48
*abbotti*	-0.27	-0.12	-0.09	-0.04	-0.50	-0.28	-0.18	-0.29	-0.86	-0.65	-0.51	-

Values greater than zero(bold font) indicate that specimens are diagnosable from one another under the rule that ≥ 75% of bill-size values for specimens of one named subspecies must fall outside the distribution of bill-size values for the other named subspecies.

Applying the diagnosability test to groups defined by broad geographic regions produced largely similar findings. Specimens from Florida the Bahamas and the Greater Antilles were reciprocally diagnosable from specimens from the Lesser Antilles based on bill size, but otherwise specimens from different regions were not distinguishable under the 75% rule (Table 5). Specimens composing the two groups identified in the cluster analysis of bill size were not diagnosable either (diagnosability indices = -0.01 and -0.05, respectively).

**Table 5:** Pairwise diagnosability indices of bill size for specimens of Mangrove Cuckoo (*Coccyzus minor*) collected in different geographic regions.

	Region of origin					
	Florida and the Bahamas	Greater Antilles	Antigua and Barbuda	Lesser Antilles	South America	Middle America
Florida and the Bahamas	-	-0.50	-0.23	**0.05**	-0.50	-0.30
Greater Antilles	-0.47	-	-0.10	**0.12**	-0.44	-0.88
Antigua and Barbuda	-0.22	-0.78	-	-0.23	-0.79	-0.56
Lesser Antilles	**0.09**	**0.21**	-0.18	-	-0.50	-0.24
South America	-0.29	-0.16	-0.55	-0.31	-	-0.72
Middle America	-0.36	-0.18	-0.49	-0.22	-0.78	-

Values greater than zero (bold font) indicate that specimens are diagnosable from one another under the rule that ≥ 75% of bill-size values for specimens of one named subspecies must fall outside the distribution of bill-size values for the other named subspecies.

None of the putative subspecies were reciprocally diagnosable under the 75% rule when applied to breast color (Fig 6). The palest forms, *C*. *m*. *maynardi*, *C*. *m*. *caymanensis*, and *C*. *m*. *grenadensis*, were all diagnosable from the rather dark *C*. *m*. *dominicae* (color scores for 87%, 84%, and 81% of the specimens of *C*. *m*. *maynardi*, *C*. *m*. *caymanensis*, and *C*. *m*. *grenadensis*, respectively, were outside of the range of color scores observed for *C*. *m*. *dominicae*), but *C*. *m*. *dominicae* was not diagnosable from any of those subspecies. Overlap in breast color among specimens grouped by broad geographic region was even greater (Fig 6) and none were diagnosable from one another based on breast color.

## Discussion

Conclusions about the nature of phenotypic variation within the Mangrove Cuckoo, notably in bill size and ventral color, have been divergent. Many authors believed that this highly plastic species could be subdivided into numerous well-defined subspecies, whereas Banks & Hole [3] argued for a total lack of geographic structure in phenotype and advocated treating the species as monotypic. The findings presented here suggest that the truth lies somewhere between these two extremes.

On one hand, I found little empirical support for recognizing any of the previously defined subspecies of Mangrove Cuckoo. None of the putative subspecies that were recognized historically were reciprocally distinguishable from all other subspecies when I applied the 75% rule [18] to bill size or ventral color, supposedly defining traits identified by authors of most of the named subspecies. Although each of the four putative subspecies from Florida and the Bahamas and the Greater Antilles was diagnosable from each of the three subspecies from the central Lesser Antilles, none of the putative subspecies within each region were diagnosable from one another, and none were diagnosable from the putative subspecies occupying South and Middle America, including the nominate form *C*. *m*. *minor*.

In addition, despite fairly pronounced differences in bill size between the subspecies of the central Lesser Antilles and those from Florida, the Bahamas, and the Greater Antilles, these two groups of putative subspecies were separated geographically by individuals on Antigua and Barbuda (*C*. *m*. *rileyi*) that were intermediate in bill size, suggesting that phenotypic variation was clinal rather than discrete. The clinal nature of variation in bill size in this species was even more evident when examining geographic variation irrespective of previously defined subspecific boundaries. Although the extremes of this cline were quite distinctive, both were indistinguishable from the host of intermediate forms. As such, variation in bill size of Mangrove Cuckoo appeared more as a continuous function than a categorical phenomenon that would lend itself to recognition of subspecies.

Analysis of ventral color supported the interpretation of phenotypic variation in Mangrove Cuckoo as clinal rather than discrete. None of the subspecies were reciprocally diagnosable based on ventral color, but populations became progressively darker from Florida and the Bahamas through the Greater Antilles, reaching a maximum in the Lesser Antilles on Dominica before fading again in collections from St. Vincent, Grenada, and South and Middle America. Variation in ventral color was not entirely concordant with variation in bill size, however; many specimens from the small-billed populations of the Greater Antilles were relatively dark (e.g., birds from Jamaica, Hispaniola, and Puerto Rico) whereas the large-billed birds from Grenada were relatively pale, often indistinguishable in color from specimens collected in Florida and the Bahamas. In addition, as has been noted before [3], relatively dark and relatively pale specimens could be found in any of the putative subspecies and at any given collection locale. As with bill size, distinguishing among the extremes of ventral color was relatively straightforward–a typical specimen from Dominica was unlikely to be confused with a typical bird from Florida–whereas discerning regular geographic patterns of variation among the intermediate forms was impossible. Ventral color varies widely among individuals, but not in a way that suggests that it would be useful in differentiating among most populations.

Caution is also warranted in interpreting the results of the analysis of ventral color due to the possible confounding effect of variation in collection date. The intensity of plumage coloration appears at least somewhat dependent on collection date, due to effects of seasonal feather wear [10,21], and within the sample of birds that I examined collection date differed among subspecies. Thus, any real differences in color among subspecies may have been confounded by differences in feather wear. For example, most specimens of *C*. *m*. *nesiotes*, often described as one of the more richly colored subspecies, were collected between November and March, when plumage should be relatively fresh assuming an annual molt that follows a breeding season probably ending in July [6]. In contrast, the majority of specimens of *C*. *m*. *maynardi*, considered one of the palest subspecies, were collected between May and July, when plumage has yet to be refreshed by the post-breeding annual molt and is thus at its most worn and faded. Further complicating interpretation of plumage color is that timing of molt may vary widely, both among populations [6] and within populations [22], such that specimens collected in the same locale at the same time may be at very different stages in their annual cycle.

Although the proposed subspecies were not diagnosable based on bill size or ventral color, geographic variation in phenotype did not seem random, as has been argued by Banks & Hole [3]. They argued that any differences in color or morphology among populations were the result of founder effects, and furthermore that these differences were not stable because other phenotypes would invade over time and, presumably by chance, come to dominate. Random variation of this sort is unlikely to yield regional clustering in bill size as was observed in these data. Especially at odds with the notion of unstructured phenotypic variation was the relatively abrupt change in bill size between the Greater and Lesser Antilles, with only several hundred kilometers separating the small-billed population on Tortola from the large-billed population on Montserrat. The steep slope of the bill-size cline around the islands of Antigua and Barbuda suggests that phenotypic variation in this trait is not random, but may instead reflect an adaptive response to a categorically varying factor or to a steep gradient in some continuously varying feature of the environment.

For example, differences in bill size might reflect intensity of competition from congeners [23–25]. Mangrove Cuckoos co-occur with one to two much larger cuckoo species on each island from the Bahamas through the Greater Antilles [26], but on the relatively species-poor islands of the Lesser Antilles they are the only cuckoo present. Interactions between Mangrove Cuckoos and their larger relatives are poorly documented, but there appears to be broad overlap in both habitat [27,28] and diet [29,30]. In one study, abundance of Mangrove Cuckoo was substantially lower where the species co-occurred with a larger congener [28]. In the absence of larger competitors, larger bill size may be favored because it provides access to a greater range of food resources [25], for example the *Anolis* lizards that can form a large part of the species’ diet in the Lesser Antilles [31]. The intermediate bill size among birds on Antigua and Barbuda, on which Mangrove Cuckoo is the only resident cuckoo, may reflect high levels of gene flow from Greater Antillean populations. What might favor the intermediate distribution of bill sizes throughout the rest of the range is unclear, primarily because the community ecology of Mangrove Cuckoo in South and Middle America is poorly known [6].

However, several other species with a geographic range that includes islands of both the Greater and Lesser Antilles show a similar pattern of larger bill size among subspecies or populations in the Lesser Antilles, which suggests a more general phenomenon driven by climate or another large-scale environmental gradient. The subspecies of Gray Kingbird (*Tyrannus dominicensis*) that inhabits the Greater Antilles (*T*. *d*. *dominicensis*) has a substantially smaller bill than the Lesser Antillean form (*T*. *d*. *vorax*); the same pattern is evident among subspecies of Antillean Euphonia (*Euphonia musica*) [32,33]. Adelaide’s Warbler (*Setophaga adelaidae*), Barbuda Warbler (*S*. *subita*), and St. Lucia Warbler (*S*. *delicata*), once treated as populations of a single species, also show an increase in bill size from Puerto Rico to Barbuda to St. Lucia [34]. A potentially fruitful avenue for improving our understanding of phenotypic evolution among Antillean birds would be to document the extent to which different species show correlated changes in phenotype across a geographic gradient from the Greater to Lesser Antilles, and whether these changes co-vary with measurable environmental gradients.

The existence of a geographic pattern of variation in bill size, and to a lesser extent ventral color, suggests that Mangrove Cuckoo is not as panmictic as has been argued [3]. The absence of any large-billed individuals (i.e., like those found in the central Lesser Antilles) in collections I examined from Florida, the Bahamas, or the Greater Antilles, which span more than 100 years of sampling effort (1877–1986), suggests that long-distance dispersal between islands and populations is not a frequent and regular occurrence. Mangrove Cuckoos are evidently capable of long overwater flights, as evidenced by their presence on nearly every island in the West Indies, but there was little evidence in these data to indicate that “populations or individuals frequently move from one island to another” [3]. The lack of large-billed individuals collected in the Greater Antilles also argues against the importance of wind as the key facilitator of long-distance dispersal. Prevailing winds, and those associated with tropical cyclones, generally blow from east to west, which should facilitate movement of individuals from the Lesser Antilles to the Greater Antilles. If wind is the main agent of dispersal, then large-billed individuals should regularly appear in the Greater Antilles, yet I found no evidence that they do. Indeed, to the contrary, if there was any pattern of vagrancy notable in these data, it was that of relatively small-billed individuals appearing in some Lesser Antillean populations (notably St. Vincent and Grenada), presumably without the aid of prevailing winds.

Naturalists have long noted the extensive variability in the appearance of Mangrove Cuckoos, but have drawn varying conclusions about the nature of that variation. The results presented here suggest that this variation is neither as regular and discrete nor as entirely random as has been argued at various times. Geographic patterns of variation are evident, but the variation is clinal, such that recognizing subspecies is not a useful way of understanding phenotypic variation. However, the slope of the cline varies substantially across the geographic range of the species, as would be expected if different populations were exposed to different selection pressures. Mangrove Cuckoo may therefore prove useful as a model for testing hypotheses concerning the adaptive value of variation in bill size [35]. Finally, in addition to informing our understanding of taxonomy and identifying useful avenues for future research, these results also demonstrate the ecological insights possible from descriptive studies of phenotypic variation

## Supporting Information

S1 Photograph Specimens of Mangrove Cuckoo (*Coccyzus minor*) arranged geographically to represent general geographic patterns in ventral color. Specimens from Florida and the Bahamas and the Cayman Islands (uppermost left specimen; *C*. *m*. *maynardi*) were palest. Continuing clockwise, ventral color gradually darkened from birds of the Greater Antilles (*C*. *m*. *nesiotes*) to birds of Antigua and Barbuda (*C*. *m*. *rileyi*) to the very dark forms of the central Lesser Antilles (*C*. *m*. *dominicae* and *C*. *m*. *vincentis*, respectively). Specimens from Grenada (*C*. *m*. *grenadensis*) were again lighter in color, and were similar to specimens from South America (*C*. *m*. *minor*; both specimens) and Middle America (*C*. *m*. *palloris*; both specimens shown), including offshore islands (*C*. *m*. *abbotti*). Not all subspecies shown.
(TIFF)

S2 Photograph Comparison of ventral color between a typically light subspecies and a typically dark subspecies of Mangrove Cuckoo (*Coccyzus minor*). Specimens with relatively light or relatively dark ventral plumage can be found among any of the named subspecies of Mangrove Cuckoo. For example, although specimens of *C*. *m*. *maynardi* were generally pale (typical specimen far left), some individuals were as dark (second from left) as relatively pale specimens of *C*. *m*. *dominicae* (second from right), which was generally the darkest of the named subspecies (typical specimen far right).
(TIFF)
