# Supplementary figures and images for: Phenotypic Variation in Mangrove Cuckoo (Coccyzus minor) across Its Geographic Range
Source: PLoS One. 2016 Mar 23;11(3):e0152141. doi: 10.1371/journal.pone.0152141 (PMC4805237; doi:10.1371/journal.pone.0152141)

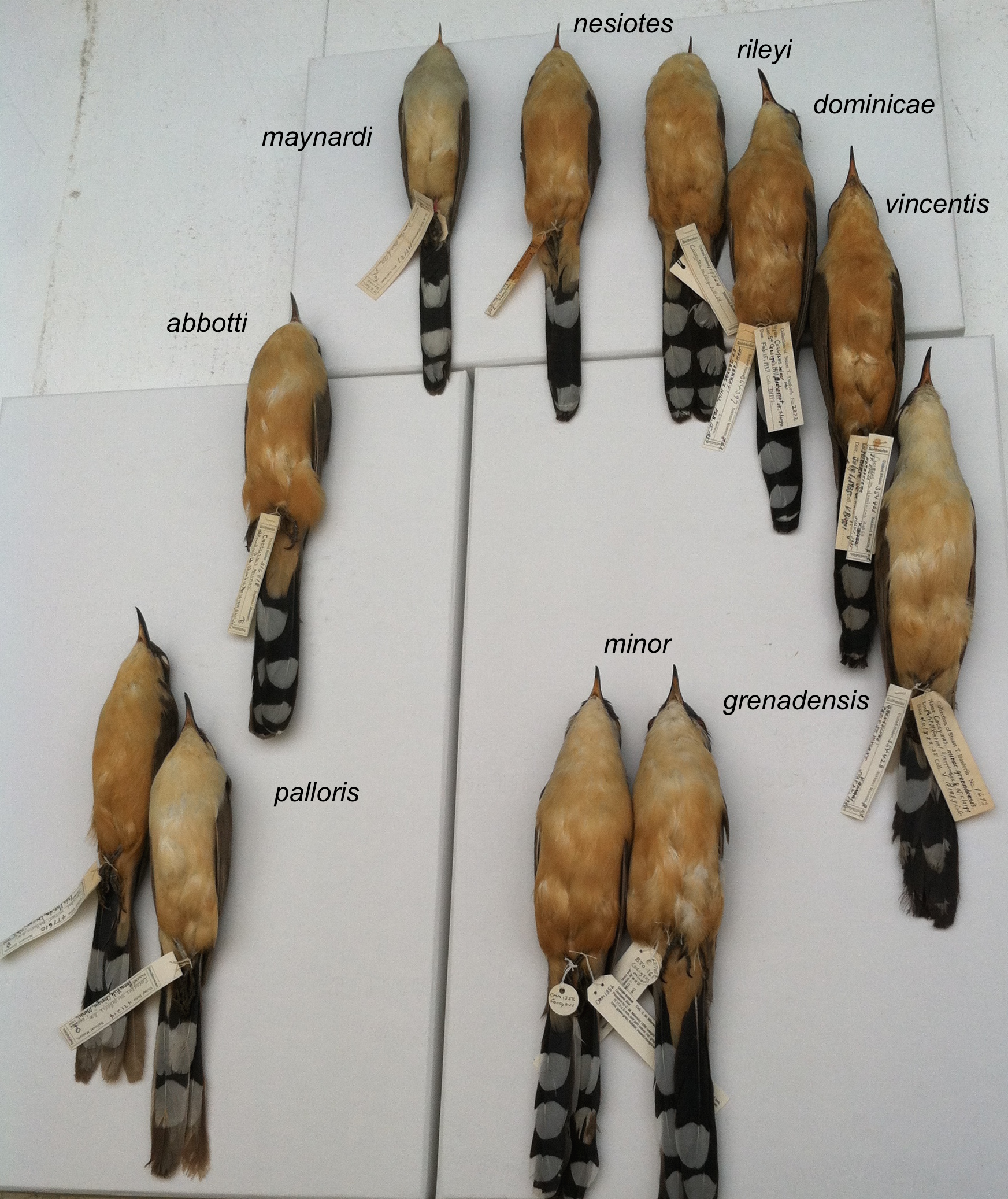

Supplement: S1 Photograph — Specimens from Florida and the Bahamas and the Cayman Islands (uppermost left specimen; C. m. maynardi) were palest. Continuing clockwise, ventral color gradually darkened from birds of the Greater Antilles (C. m. nesiotes) to birds of Antigua and Barbuda (C. m. rileyi) to the very dark forms of the central Lesser Antilles (C. m. dominicae and C. m. vincentis, respectively). Specimens from Grenada (C. m. grenadensis) were again lighter in color, and were similar to specimens from South America (C. m. minor; both specimens) and Middle America (C. m. palloris; both specimens shown), including offshore islands (C. m. abbotti). Not all subspecies shown. (TIFF) [file pone.0152141.s001.tiff]

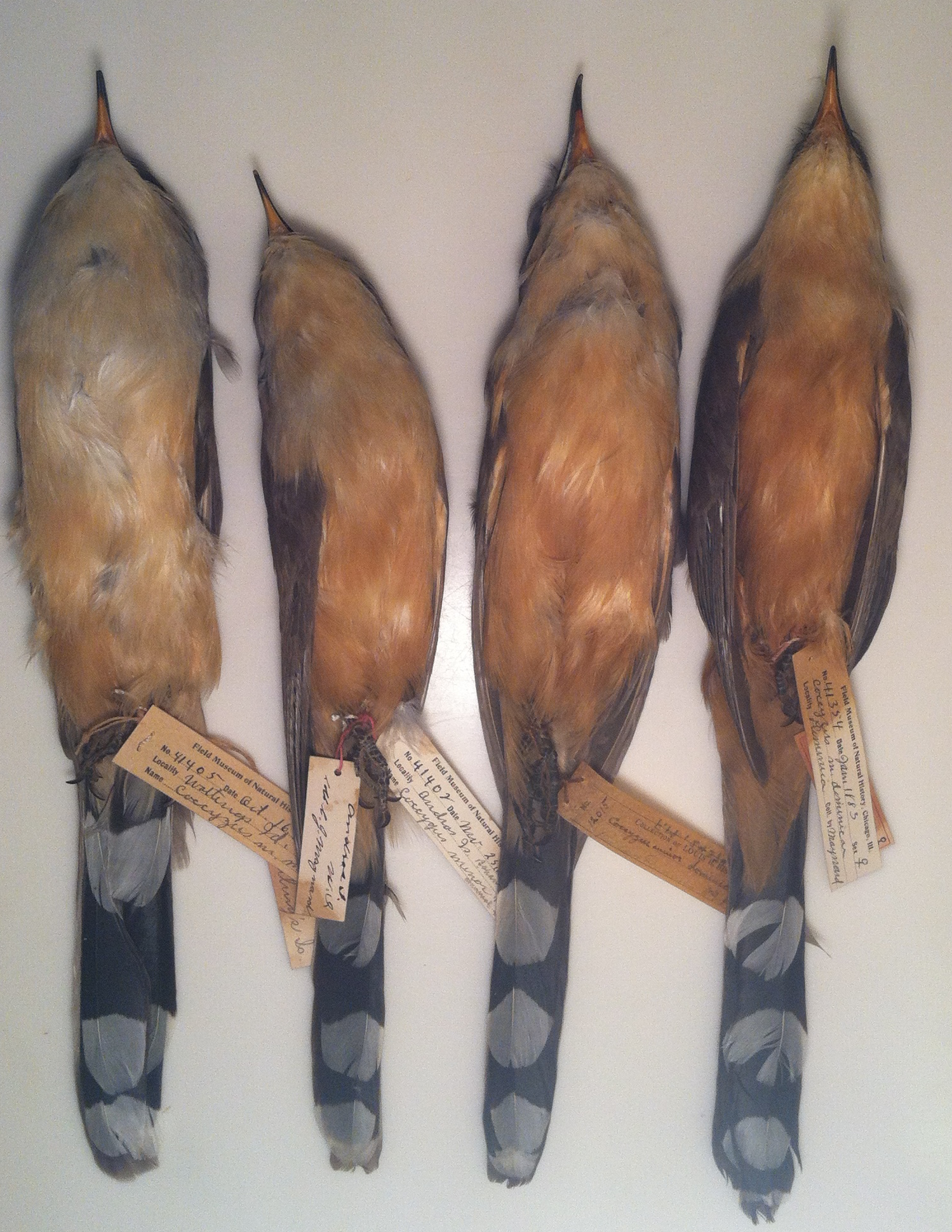

Supplement: S2 Photograph — Specimens with relatively light or relatively dark ventral plumage can be found among any of the named subspecies of Mangrove Cuckoo. For example, although specimens of C. m. maynardi were generally pale (typical specimen far left), some individuals were as dark (second from left) as relatively pale specimens of C. m. dominicae (second from right), which was generally the darkest of the named subspecies (typical specimen far right). (TIFF) [file pone.0152141.s002.tiff]
